# Patient Satisfaction with Spanish Pain Centers: Observational Study with More than 3,000 Patients

**DOI:** 10.1155/2016/7829585

**Published:** 2016-07-19

**Authors:** Juan Antonio García García, Patricia Hernández-Puiggròs, Javier Tesedo Nieto, María Pilar Acín Lázaro, Alfredo Carrera González, Miguel José Arranz Soler, Sergio Maldonado Vega

**Affiliations:** ^1^Pain Unit, Hospital Infanta Cristina, 28981 Madrid, Spain; ^2^Pain Unit, Hospital Son Llàtzer, 07198 Palma de Mallorca, Spain; ^3^Pain Unit, Hospital Clínico Universitario de Valladolid, 47003 Valladolid, Spain; ^4^Pain Unit, Hospital Royo Villanova, 50015 Zaragoza, Spain; ^5^Pain Unit, Hospital Universitario Marqués de Valdecilla, 39008 Santander, Spain

## Abstract

Chronic pain is a serious problem in Spain. This multicenter, epidemiological 3-month follow-up study investigates pain management efficacy in Spanish centers using patient satisfaction criteria. 3,414 eligible adult patients (65,6% female) with moderate to severe chronic pain from 146 pain centers were included. Patient satisfaction was assessed based onto question 18 of Spanish healthcare barometer-CSI. Pain evolution (Brief Pain Inventory-Short Form (BPI-SF) and visual analog scale (VAS)), quality of life/EuroQol-5, and pain control expectations fulfillment were also assessed. Mean age was 61.3 years. 64.4% of participating centers employed multidisciplinary pain management approach. After 3 months, mean patient satisfaction was 7.8 (1–10) on the CIS barometer. Medical staff received the highest scores, whereas waiting for tests, appointment request to appointment date time, and waiting times at the center the lowest. Mean pain decreased from 7.4 to 4.0; BPI-SF intensity decreased from 6.5 to 3.8; pain control expectations were met in 78.7% of patients; EuroQoL-5D utility index increased from 0.37 to 0.62, *p* < 0.001, and health status (VAS) from 40.6 to 61.9, *p* < 0.001. Chronic pain patients (90%) are satisfied with Spanish centers care; 80% had their pain control expectations met. Quality of life improved remarkably: 71% felt moderately to significantly better. However, waiting times need improvement.

## 1. Introduction

Chronic pain is a serious public health problem in Spain. It has a prevalence of 23.4% in the Spanish general population and important health and economic repercussions [1]. The 1-year prevalence of neck, low back pain, and migraine in Spain is 19.5%, 19.9%, and 11.02%, respectively [2, 3], whereas the prevalence of incapacitating musculoskeletal pain is 6.4% [4]. All the above pain conditions occur more frequent in women than men and are associated with worse health status, depression, and other comorbidities [2, 3, 5]. Chronic pain is also very prevalent among cancer patients, ranging from 33% to 64% according to disease stage [6] and becomes a long-standing issue in many occasions [7, 8]. In a high proportion of chronic pain cases, 33% of cancer patients with pain and 46% of patients with noncancer pain in primary care [9–11], pain is neuropathic (NP) in origin. These patients with NP have the highest prevalence of insomnia, anxiety, depression, severity of pain, and also the highest healthcare-related costs among all the patients with chronic pain [12, 13].

Controlling pain becomes increasingly difficult as pain becomes chronic [14]. In addition to its association with other psychiatric and physical comorbidities (anxiety, depression, insomnia, etc.) [15], pain has an important emotional component and is associated with negative affection states, such as fear [16–18]. Moreover, cultural and environmental influences and stimuli may also affect the perception of pain [19]. Taking into account all these factors, chronic pain patients could benefit by having their condition managed in a specialized pain centers [18, 20–22], where, in addition to classical analgesic treatments, cognitive-behavioral treatments may be employed [23].

Inappropriate pain management is associated not only with increased patient suffering but also with great financial costs, in terms of loss of work time, reduced levels of productivity, and ability to function in society [24]. In Spain, previous studies have shown that a large percentage of the resources allocated by the National Health Service to fund physical treatment for back, neck, and shoulder pain in private practices are spent on treatments proven to be ineffective, or there is no clear evidence that they offer any benefits to the patients [25].

However, studies on the efficacy of pain centers or pain units in Spain are scarce.

Information regarding the effectiveness of pain management is based more on experience from routine clinical practices rather than on studies measuring the efficacy of a single drug treatment or therapeutic intervention [26]. Patient-reported outcomes (PROs) include any outcomes based on data provided by the patients and are very important for understanding the impact of treatment on patient functioning and wellbeing [27]. Healthcare results are measured in terms of satisfaction with the achieved outcome and in the literature there are studies on patient satisfaction with hospitalization or follow-up visits. On the other hand, there are hardly any studies on patient satisfaction with medical outcomes, which can be better related to clinical use [28].

The high prevalence of chronic pain with its associated allocation of resources and corresponding economic impact justifies the need to assess the effectiveness of patient management in the Spanish pain centers. Measurement of patient satisfaction was the selected method to evaluate the centers in terms of effectiveness and to identify existing gaps in management that could be further analyzed and improved.

## 2. Methods

A 3-month follow-up prospective, longitudinal, multicenter, descriptive, and epidemiological study was conducted among the participating Spanish pain centers. Pain observational studies in general have short follow-up times. A 3-month follow-up time was considered sufficient for valid conclusions. Patients' degree of satisfaction with such centers was assessed by means of a questionnaire, based onto the question 18 of the Spanish healthcare barometer or CIS barometer, which is related to the healthcare service. As secondary objective, we assessed the evolution of pain, according to the Brief Pain Inventory-Short Form (BPI-SF) and to a visual analog scale (VAS), and the quality of life (QoL) as well, according to EuroQol-5, in patients followed up in the pain units.

The target pain, as estimated by the participating researchers, was 2.2 based on a VAS. The target pain of 2.2 is the median value calculated for each patient by the participating physicians, according to the clinical condition of the patient and the experience of the physician. In addition, the following variables were assessed: patient satisfaction with pain control by means of a VAS, patient global clinical impression of change (PGCI-C), and compliance with patients expectations regarding pain control, based on the Patients Expectations Questionnaire (PEQ) [29].

### 2.1. Study Design

The study protocol was carried out in accordance with the declaration of Helsinki (Seoul, 2008). Spanish regulations were taken into account as well, including approvals by the Ethics Committee for Clinical Research of the University Hospital of Getafe (registration number E-08/018), and all other relevant Ethics Committees, as it was deemed necessary for the evaluation of the patients.

One hundred and seventy-three investigators from 146 centers throughout the Spanish national territory participated in the study. To avoid selection bias, inclusion and exclusion criteria were established. The inclusion criteria in the study were as follows: every participating researcher recruited the first 10 to 30 adult patients over the age of 18 that visited the outpatient during the agreed study period. The pain intensity was ≥4 (according to a 1–10 VAS), and the patients were visiting the pain center for the first time. Pain referrals (somatic, visceral, and neuropathic) from all clinical specialties, all social backgrounds, and working status were included (Table 1). Informed consent was obtained from all participating patients. The exclusion criterion was psychiatric or neurologic disorders, which could affect the patient perception of pain, as it was judged by the treating physician assessment. Patients were enrolled in the study from September 2011 to March 2012. The follow-up period ended in June 2012.

The following data were collected: medical specialties and analgesic techniques available at the pain unit; sociodemographic and clinical data of the patients, including pain origin, types of pain, and baseline pain, which was classified as moderate-intense (VAS < 7) and very intense (VAS ≥ 7). In addition, the following data were also collected at 3 months: intensity of pain and its impact on activities of everyday living (BPI-SF); current pain intensity (VAS) as assessed by the physician; quality of life (EuroQoL-5). At the 3-month follow-up visit, the following data were collected: patient satisfaction with pain control (VAS), fulfillment of expectations regarding pain control (PEQ), patient's impression of change in pain (PGCI-C), and patient satisfaction with the pain unit (CIS barometer).

The mean pain intensity (VAS) and total pain interference reductions were divided into two variable categories (mean pain reduction <30% and ≥30% and pain interference reduction <30% and ≥30%), according to the percentage of change, following the definition of “moderately important improvement” by the Initiative on Methods, Measurement, and Pain Assessment in Clinical Trials (IMMPACT) group [30]. This group has defined the parameters and outcomes, which should be assessed in chronic pain clinical trials (pain, physical functioning, emotional functioning, participant ratings of improvement and satisfaction with treatment, symptoms and adverse events, and participant disposition), and provided the relevant recommendations to determine the clinical importance of change in these outcome measurements. Regarding pain intensity, assessed by a 0 to 10 numerical rating scale, a 10–20% decrease in pain intensity was considered as a minimally important improvement, a decrease ≥30% as a moderately important improvement, and a decrease ≥50% as a substantial improvement according to the main IMMPACT outcome recommendations [30].

### 2.2. Scales and Questionnaires

The Spanish healthcare barometer or CIS barometer is an annual opinion survey conducted since 1995 by the Spanish Ministry of Health (Ministerio de Sanidad y Política Social) in collaboration with the Sociological Investigation Center (CIS). Its purpose is to understand the general public perception of the public healthcare services (whether they have used them or not) [31]. The patients' degree of satisfaction with several aspects of the specialist healthcare service is presented through the answers to question 18 in 2012 [32]. In the current study, the barometer was exclusively used to measure the satisfaction of the participating chronic pain patients with the pain centers, where they were being treated, by answering all the items of question 18. Question  18 has 12 items, and each one of them should be valued by giving a score from 1 (completely unsatisfied) to 10 (totally satisfied). An average score ≥6 is considered a satisfied patient, based on data from the barometer in 2009. Usage of the barometer's data allows for a median satisfaction score of 6.35 to be calculated by averaging all the achieved median public satisfaction for the tested services [31]. Thus, the patient satisfaction with the pain unit was classified as a two-variable parameter: satisfied (score ≥ 6) and not satisfied (score < 6).

The Brief Pain Inventory (BPI) [33, 34] is a self-administered tool to assess the intensity of pain and its impact on activities in everyday living. The Spanish version has been validated [35]. The short form, which contains 11 items, was used in a recent study by de Andrés Ares et al. [36]. The items are rated on a 0 (no pain/no interference) to 10 (worst possible pain/total interference) numeric rating scale (NRS) and are grouped in two categories: pain intensity (the mean score of the first 4 items: worst, least, and average pain in the past 24 hours and pain right now) and interference with daily activities (the mean score of the last 7 items: interference with general activity, mood, walking ability, normal work, relations with others, sleep, and enjoyment of life). Pain intensity is classified as mild or no pain (0–3), moderate (4–6), and intense (≥7).

The EuroQoL-5D (EQ-5D) is a standardized, non-disease-specific instrument for describing and valuing health-related quality of life [37]. The use of the validated Spanish version [38] was approved by the EuroQoL Group Foundation. This instrument rates mobility, self-care, usual activities, pain/discomfort, and anxiety/depression and employs three severity levels (1 means no problem, 2 means some or moderate problems, and 3 means many problems). The different combinations of the EQ-D's 5 valued items provide 243 possible health status possibilities. The levels 2 (some or moderate problems) and 3 (many problems) were grouped together as “problems,” as opposed to level 1 or “no problem.” There is also a 100 mm VAS with two scale ends: the worst (0 or equivalent to death) and the best imaginable health status (100), where the patient indicates the perception of his own overall health. Another parameter of the EQ-5D is the index of social preference values, obtained for each health status from the overall population studies. The index varies from 1 (best health status possible) to 0 (death), although negative values exist corresponding to health status considered as worse than death.

The patient global clinical impression of change (PGCI-C) [39] is a self-administered scale assessing the experienced change in pain from no change to a great deal better.

The Patients Expectations Questionnaire (PEQ) [29] gathers information related to the fulfilled patients' expectations, scoring five entities regarding hospitalization. In the current study, the fulfillment of patients' expectations was focused on pain control. The possible answers were “as expected, somewhat or much more than expected, somewhat or much less than expected.”

### 2.3. Statistical Analysis

Descriptive statistics were performed for every variable, including central and dispersion measurements for continuous variables, and absolute and relative frequencies for categorical variables, with 95% confidence intervals (CIs), at baseline, 1 month, and 3 months. Missing data were not included in the analyses and were considered as lost.

Continuous variables were compared between baseline and 3 months by means of the Student's* t*-test in case of parametric data. Categorical variables were compared by the chi-square test or by nonparametric tests (Fisher exact test, *U* Mann-Whitney, Wilcoxon, etc.) for parametric and nonparametric data, respectively. For the main and secondary variables, the effect size, comparing 3 months versus baseline, was calculated according to the Cohen formula [40], using the pooled standard deviation as the denominator of the equation. Logistic regression analyses were performed to assess putative factors (sex, age, and baseline pain) that might be associated with the satisfaction with the pain unit.

Statistical tests were performed with a bilateral 0.05 significance level. The SPSS software version 17.0 was used for the statistical analyses.

## 3. Results

**Figure 1:**
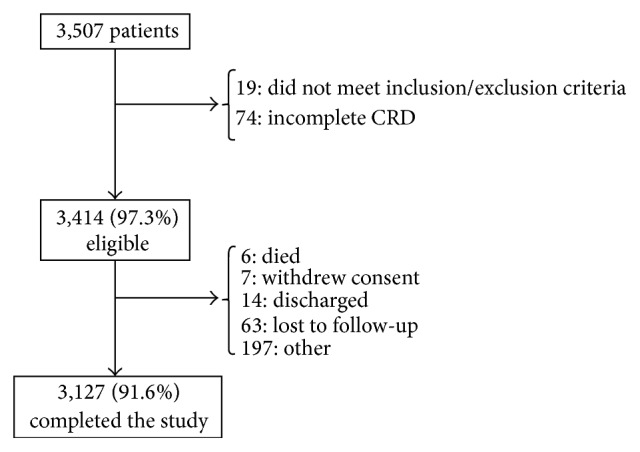
Patients flow chart.

Out of 3,507 patients initially enrolled, 93 patients were excluded: for 74 of them, there was no data available and 19 patients did not comply with the inclusion criteria. Out of the 3,414 eligible patients, 3,127 (91.6%) completed the study, 6 patients died, 7 withdrew their consent, 14 were discharged, 63 were lost to follow-up, and 197 did not complete the study due to other reasons (Figure 1).

**Figure 2:**
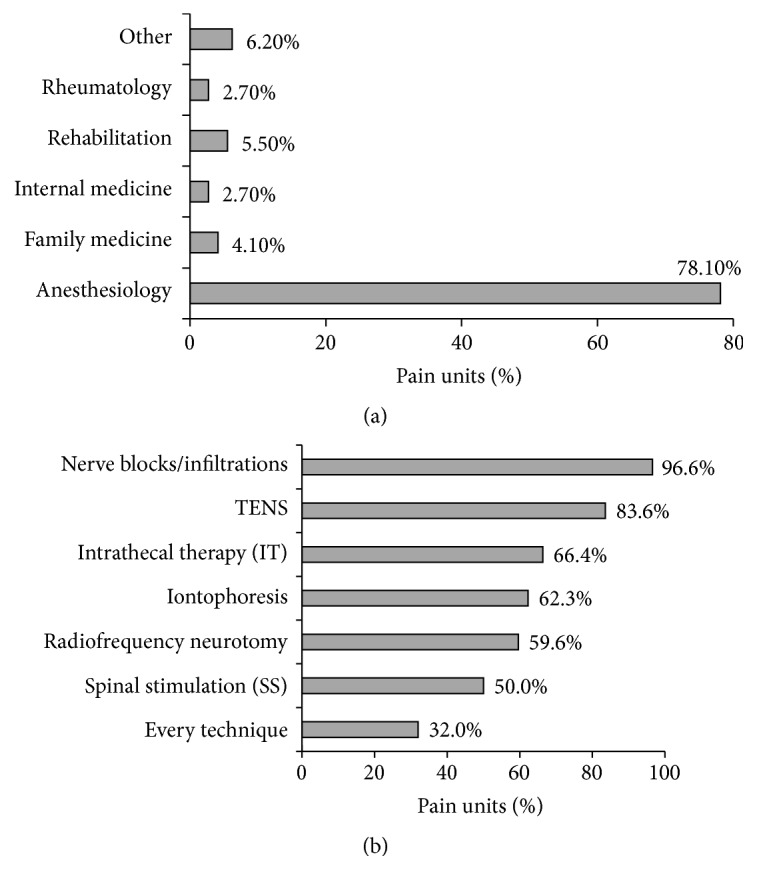
Specialties (a) and techniques (b) available in the pain centers.

Every Spanish region (Autonomous Community) had at least one pain center. Out of the 146 participating pain centers, 64.4% were multidisciplinary (more than one specialty) and 37% had at least three different specialties. In multidisciplinary pain units, medical staff from various specialties (anesthetists, psychologists, and physiotherapists) is involved in the pain management. Regarding available techniques, the most commonly used in almost every center is nerve blocking (96.6%), followed by TENS (83.6%), while spinal stimulation is provided in 50% of pain centers. Almost half of the centers (47.9%) had both spinal stimulation and intrathecal therapy techniques. One-third of the centers had every available pain controlling technique (Figure 2(b)).

Patients mean age was 61.3 years and 65.6% were female (Table 1). They were referred to the pain centers mostly from traumatology (40.6%), followed by primary care (22.5%). Most lived together with a family member (84.3%) and 22% required third-party care. Almost half of the patients were retired. About three-quarters of the patients experienced worse health status during the study period compared to the previous 12 months. Most pain was mixed in origin, and visceral pain was rare (2.4%). Main types of pain were arthritic/arthritis (55.6%) and hernia/disc pathology (42.8%). Mean pain intensity was 7.4 and the participating investigators targeted the pain intensity down to 2.2, which is, as it was mentioned earlier, the median value calculated for each patient by the participating physicians, according to the clinical condition of the patient and the experience of the physician. The most frequent areas of intense pain were the posterior lumbar (47%) and posterior sacral areas: center (49.8%), left side (44.9%), and right side (44.4%), followed by the posterior upper leg (41.6%) area.

### 3.1. Patient Satisfaction with the Pain Center

After three months of care, mean patient satisfaction with the pain center, as assessed by the CIS barometer, was 7.8, with more than 90% of the patients being satisfied (Table 2). The items with the highest scores were those related to the treating physicians and overall healthcare personnel, followed by the equipment and technology available at the center, while the items with the lowest scores were the waiting times for the diagnostic tests, from medical appointment request to appointment date and at the center to see the doctor, which compare similarly with the data from question 18 of the CIS barometer from 1995 to 2012 (Table 4).

### 3.2. Secondary Objectives

At three months, baseline mean pain (VAS) decreased from 7.4 to 4.0 (Table 3), and 67.4% of patients showed a mean decrease of pain intensity of at least 30% (data not shown). Accordingly, BPI-SF intensity summary also decreased from 6.5 to 3.8, while the percentage of patients feeling pain relief in the last 24 hours due to received treatment increased from 29.1% to 60.9%. The interference summary decreased from 44.8 to 26.4, and 63% of patients showed a pain interference reduction of at least 30% (data not shown).

**Figure 3:**
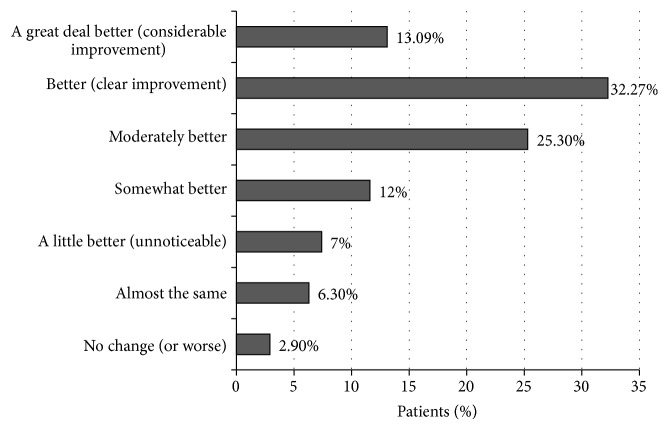
Patients global clinical impression (PGCI) of the change at month 3.

**Figure 4:**
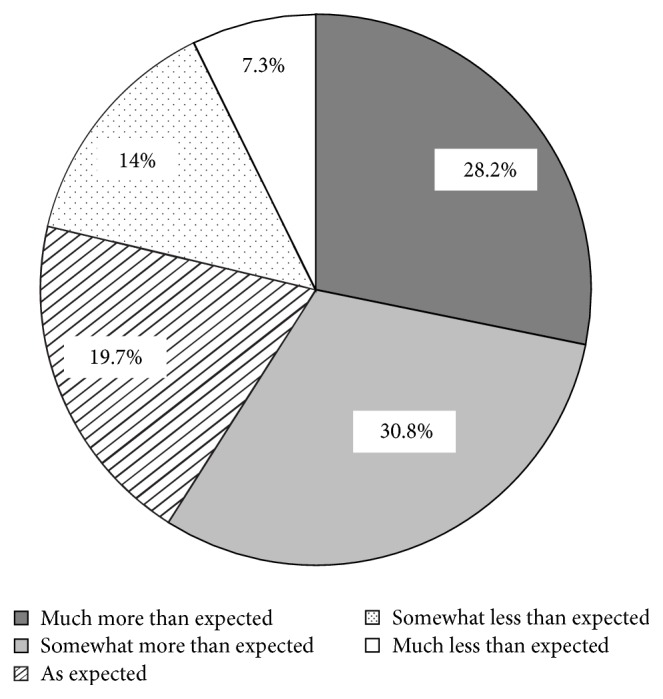
Pain control degree according to patients' expectations.

In addition, 70.7% of patients at three months felt moderately to much better, according to the PGCI (Figure 3), and mean satisfaction with pain control was 6.6 ± 2.2 in a visual analog scale (data not shown). Most patients (78.7%) felt that their expectations regarding pain control since their first visit to the pain center had been met as expected or more (Figure 4).

**Figure 5:**
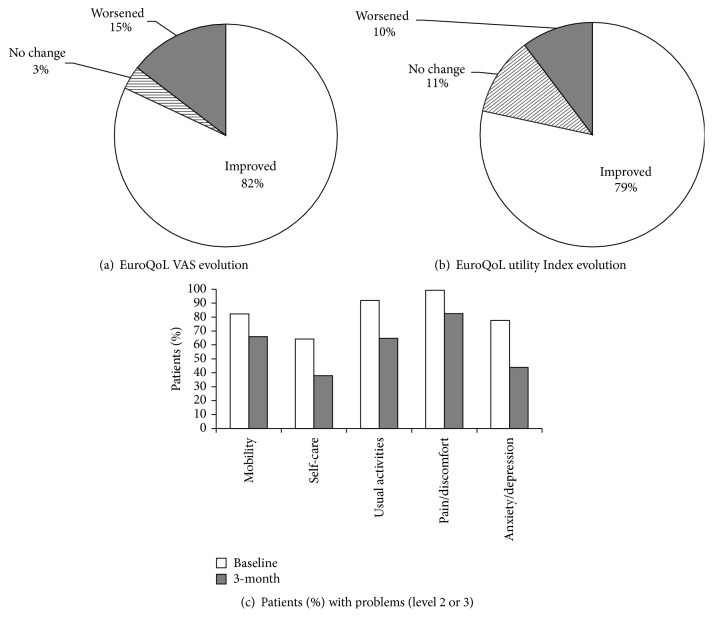
Change in EuroQoL VAS (a) and utility index (b) at 3 months and proportion of patients with problems (level 2 or 3) in the EuroQoL dimensions at baseline and 3 months (c).

Regarding quality of life, the EuroQoL-5D utility index increased from 0.37 to 0.62, *p* < 0.001 (effect size *d* = 1.19) and the health status (VAS) from 40.6 to 61.9, *p* < 0.001 (effect size *d* = 1.1) (Table 3). Eighty-two percent and 79% of patients improved in EuroQoL VAS and utility index, respectively (Figures 5(a) and 5(b)), while the proportion of patients with problems (level 2 or 3) decreased across all 5 parameters at three months (Figure 5(c)).

Logistic regression analysis showed that patient satisfaction with the pain unit (satisfied VAS ≥ 6 and not satisfied VAS < 6) was affected (*p* < 0.005) by the baseline pain intensity (moderate-intense VAS < 7 and very intense VAS ≥ 7).

## 4. Discussion

Chronic pain is a complex psychosocial entity, whose management can be very challenging [14]. Its impact on quality of life can be extremely negative [18]. Chronic pain can result in depressed mood, poor-quality or nonrestorative sleep, fatigue, reduced activity and libido, excessive use of drugs and alcohol, dependent behavior, and disability out of proportion with impairment. This combination of chronic pain and the resultant problems is what we call Chronic Pain Syndrome (CPS).

A recent European survey on noncancer chronic pain showed poor management of the condition in Spain, with more than half of patients (55%) not being satisfied with their treatment [41]. However, the current study, conducted in 146 pain centers distributed along the Spanish national territory, has shown that more than 90% of patients were satisfied with the care provided by the pain centers.

In 64% of the centers, a multidisciplinary approach to the pain management was employed. Patients managed at multidisciplinary pain centers have shown to have better outcomes when compared to those managed by nonmultidisciplinary rehabilitation, usual care, or other strategies [42–48]. Improvement in negative emotional cognitive functions seems to be the key mechanism of the observed change in the multidisciplinary treatment approach of chronic widespread pain [49]. In a study conducted with injured workers of Washington state, patients showed similar clinical outcomes, regardless of their treatment being administered in a pain center or not [50]. However, those patients had experienced more than three years of disability before their admission to the multidisciplinary program. It has been shown that treating chronic pain as early as possible is very important, and prompt treatment following injury is a significant predictor of successful return to work [51].

The most recent data of the Spanish CIS Barometer are those of 2012 [32], showing that 70.6% of the population believes that the Spanish healthcare system works pretty well to well, although some changes are needed. Comparing these general outcomes of 2012 (Table 4), the year in which our study ended, taking into account the findings of our study (Table 2), it can be seen that chronic pain patients are more satisfied with the care received in the Spanish pain centers than the overall public with the care received in the Spanish healthcare specialists services. It is important to take into account that the barometer surveys the overall population, regardless of whether or not they had used the healthcare services.

The 146 pain centers participating in the study were distributed across the entire Spanish territory. Every Spanish region (Autonomous Community) had at least one center, which is an improvement from 2002, when only 11 of the 17 Autonomous Communities had at least one [52]. In addition, 64.4% were multidisciplinary in 2012 versus 53.6% in 2002.

Patients are satisfied with the management of their painful condition at three months of treatment and studies have shown that the positive effects of the multidisciplinary approach to chronic pain persist long after the cessation of the intervention [53, 54]. It is important to point out that patients were most satisfied with the treating physicians, followed closely by the rest of the personnel. A good interaction between the patient and the service professionals is of great importance for the success of chronic pain rehabilitation [55]. A study by Trentman et al. [56] showed that time spent with the physician, thoroughness, and listening were factors associated with the patient's perception of quality of care. Thoroughness, punctuality, listening, and clear instructions were the drivers of “very good” versus “excellent” patient's perception of the overall provider quality. Such high quality patient-professional relationship has positive repercussions on treatment outcomes in the setting of a multidisciplinary rehabilitation program for chronic pain. In fact, a previous study had also shown that changes in pain was a less important predictor of treatment satisfaction, whereas the patients perception that their evaluation was complete, the satisfaction that they experienced from a detailed and accurate explanation of the therapeutic procedures, and the realization that the treatment helped them to improve their daily activity, were the strongest predictors [57].

On the other hand, the waiting time for the diagnostic tests results, from the clinic appointment date and to actually seeing the doctor once at the center, was the item with which, patients were satisfied the least. Other studies have shown a negative association between waiting time for pain clinic appointment and healthcare system grade [58]. Therefore, improvements in this area could have a significant positive effect on the opinion of patients regarding pain centers.

As expected, there were more female patients than male, since several chronic pain conditions are more prevalent in women [2–4], and, overall, the demographic characteristics of the patients agreed with the Spanish data from the recent European noncancer chronic pain survey [41]. The most frequent location of intense pain (lumbar) corresponded to the low back, which is an extremely common problem that most people experience at some point in their lives [59]. In the European survey, the commonest diagnosis was joint pain (40%), followed by back pain (32%), but patients were recruited and managed mostly by general practitioners and other specialists instead of being treated at pain centers. At three months, not only patients were satisfied with the pain center, but their clinical symptoms had improved significantly in that period of time as well. In fact, almost 80% of patients felt their expectations regarding pain control had been met as expected or more. The pain intensity VAS score decreased from a mean 7.4 to 4.0. Although it did not reach the desired target of 2.2, the mean change (3.4) greatly exceeded the minimal important change, which, by consensus, is 1.5 in patients with low back pain [60]. A previous study conducted with Spanish chronic pain patients suffering motor disability and subjected to an intensive multidisciplinary treatment of 4 weeks in duration showed a similar significant improvement in pain intensity VAS points, from 7.4 to 3.2 (*p* < 0.01) [61].

As it was discussed already BPI-SF intensity summary decreased from 6.5 to 3.8 and interference summary decreased from 44.8 to 26.4. Sixty-three percent of patients showed a pain interference reduction of at least 30%. The BPI-SF is used to evaluate the severity of a patient's pain and the impact of this pain on the patient's daily functioning. The psychometric properties of the tool have been analyzed with acceptable reliability in various populations suffering from cancer and noncancer related pain. Various studies have shown that a two-factor model has better validity for noncancer pain patients [62, 63], as opposed to the three- or one-factor approach.

The quality of life of the patients also improved remarkably as it was tested by the Spanish version of the EQ-5D, which is a simple, valid, and practical measure and can be used as an outcome variable for research purposes and in the allocation of resources. Its ability to discriminate between healthy population and chronic patients is considered to be good [38]. The mean utility index and health status were higher than one standard deviation compared with their respective mean values at baseline; these effect size values (utility index *d* = 1.2 and health status *d* = 1.1) are quite significant, according to Cohen's results for effect size interpretation (large *d* ≥ 0.80) [40]. Seventy-one percent of the patients felt moderately to much better (PGCI), which is a higher proportion than the 53% reported among the chronic low back pain patients by a long term study, which also showed that patients are doing better or much better regarding their general wellbeing after completing a multidisciplinary rehabilitation program [53].

Various other studies have shown that in general Spanish patients are satisfied with their pain management. Malouf et al. conducted a study with the aim to document the satisfaction with pain management in a Spanish inpatient population. The study showed that patients were satisfied with the received treatment, even when they were in pain, and that patient dissatisfaction was related to the pain intensity and satisfaction with caregivers [64]. On the other hand, an epidemiology survey of chronic nonmalignant pain in Spain showed that a significant percentage of patients might be inadequately treated. In this survey, it was stated that up to 60% of patients are dissatisfied with their treatment. They concluded that the variability in the collected data reflects an inconsistency in the definition of the condition and the measurement approach to assess its impact [65].

In a survey of chronic pain in Europe across 16 countries (Spain included), interesting differences between countries were observed, possibly because of different cultural backgrounds and local therapeutic preferences. The authors concluded that chronic pain occurs in 19% of adult Europeans with serious effects on the quality of their lives. Only few patients were managed by pain specialists, nearly 50% were treated inadequately and about 60% were satisfied with the effect of treatment [66]. When patients were asked to respond to the question “would you say your pain is being adequately controlled,” 33% of the Spanish responders replied “no” and 67% “yes,” which compares equally to the findings of this study.

## 5. Conclusions

In conclusion, chronic pain patients are satisfied with the management provided by Spanish pain centers more than the overall population with the hospital specialist services. Ninety percent of patients were satisfied with the management of their pain. Almost 70% of patients experienced a decrease of their pain intensity and a pain interference reduction of at least 30%. They said that at three months they felt better and that their pain control expectations were met.

There is some area for improvement regarding waiting times: time needed for diagnostic tests, time from medical appointment request to appointment date, and waiting time at the center until seeing the doctor. Waiting time shortening will provide better care to patients and higher rates of satisfaction. Many healthcare institutions use methods like revamping of the front-line scheduling process, incorporation of patient preferences, considering alternate ways of care delivery, and making the reduction of waiting times part of the hospital's culture.

Chronic pain management can have important economic repercussions. Pain management in a multidisciplinary setting may decrease the chronic pain-associated costs, since it can reduce pension expenditures, sick leave days, and usage of healthcare resource. The care provided by the Spanish pain centers seems to be successful, and, thus, although expensive, it might save costs in the long run, which should be the objective of another study.

## Figures and Tables

**Table 1:** Baseline sociodemographic and clinical characteristics of the patients.

	*N* = 3,414
*Women*	2,239 (65.6)

*Age*	61.3 ± 14.4

*Current pain (VAS)*	7.4 ± 1.5

*Referral from*	
Primary care	768 (22.5)
Rheumatology	284 (8.3)
Traumatology	1,387 (40.6)
Internal medicine	79 (2.3)
Neurosurgery	306 (9.0)
Rehabilitation	221 (6.5)
Surgery	73 (2.1)
Other	296 (8.7)

*Living*	
Alone	425 (12.4)
With family member	2,878 (84.3)
Nursery home	71 (2.1)
Other	40 (1.2)

*Care required from a third party*	748 (22.0)

*Ambulance use for medical visits*	148 (4.3)

*Working status*	
Able to work	801 (23.5)
Retired	1,672 (49.0)
Unable to work	452 (13.2)
Medical leave of absence	488 (14.3)

*Pain origin* ^*∗*^	
Somatic	2,681 (78.5)
Visceral	81 (2.4)
Neuropathic	1,996 (58.5)

*Pain type*	
Arthrosis/arthritis	1,867 (55.6)
Hernia/disc pathology	1,435 (42.8)
Neuropathy	527 (15.7)
Osteoporosis	456 (13.6)
Myofascial pain syndrome	418 (12.5)
Other	126 (3.8)
Visceral pain	61 (1.8)
Vascular pain	62 (1.8)
Failed back surgery syndrome	47 (1.4)
Other postsurgical pains	37 (1.1)
Vertebral compression fracture	27 (0.8)
Other posttrauma pains	25 (0.7)

*Current health status as compared to last 12 months*	
Better	126 (3.7)
Same	788 (23.1)
Worse	2,499 (73.2)

^*∗*^Multiple answer question. Data expressed as *n* (%) or mean ± SD.

**Table 2:** Patient satisfaction with the pain center at 3 months (PC) and satisfaction with the Spanish healthcare specialists' service (SH) in 2012.

Items	PC	SH 2012
Time spent by the physician with you	**8.6 ± 1.3**	6.72 ± 2.00
Number of specialists to whom you have access	7.6 ± 1.8	7.64 ± 1.82
Waiting time at the center until seeing the doctor	6.9 ± 2.2	5.72 ± 2.11
Knowledge of your medical history and follow-up of your health-related problems	**8.3 ± 1.4**	6.83 ± 2.04
Confidence and trust in your doctor	**8.6 ± 1.3**	7.29 ± 2.01
Easiness to get an appointment	7.0 ± 2.3	5.72 ± 2.47
Equipment and technological means available at the center	8.0 ± 1.6	7.54 ± 1.75
Manners of healthcare personnel	**8.7 ± 1.2**	7.42 ± 1.80
Information received about your health problem	**8.4 ± 1.4**	7.30 ± 1.95
Medical advice on diet, exercise, smoking, alcohol, and so forth	8.0 ± 1.6	7.13 ± 2.17
Time from medical appointment request to appointment date	6.9 ± 2.2	4.94 ± 2.39
Time taken by the diagnostic tests	6.5 ± 2.2	5.04 ± 2.38

Total satisfaction^*∗*^	7.8 ± 1.2	
Not satisfied (<6)	200 (8.4%)	
Satisfied (≥6)	2,168 (91.6%)	

Sanitary barometer 2012 (total, three waves). Executive management of public health, quality, and innovation. Ministry of Health, Social Services and Equality and Sociological Investigation Center (CIS), Madrid, 2013.

^*∗*^Average score of the 12 items.

Data expressed as mean ± SD (continuous variables) or *n* (%) (categorical variables).

Scores ranging from 0 (no satisfaction at all) to 10 (completely satisfied).

In bold: scores above 8.0.

**Table 3:** Evolution of pain (VAS and BPI-SF) and quality of life (EuroQoL-5D).

	Baseline	3 months	*p* value
*Current pain (VAS)*	7.4 ± 1.5	4 ± 1.9	—

*BPI-SF*			
BPI-SF intensity summary	6.5 ± 1.4	3.8 ± 1.9	—
Pain relief in the last 24 h by received treatment (%)	29.1% ± 19.9%	60.9% ± 22.7%	—
BPI-SF interference summary	44.8 ± 12.5	26.4 ± 15.1	—

*EuroQoL-5D*			
Utility index	0.37 ± 0.21	0.62 ± 0.22	<0.001
Today's health status (VAS)	40.7 ± 20.1	61.9 ± 19.3	<0.001
Patients with problems (2 and 3) in			
Mobility	2,805 (82.2)	2,127 (65.9)	—
Self-care	2,194 (64.2)	1,224 (37.9)	—
Usual activities	3,142 (92.0)	2,091 (64.8)	—
Pain/discomfort	3,389 (99.3)	2,657 (82.4)	—
Anxiety/depression	2,650 (77.6)	1,415 (43.9)	—

Data expressed as mean ± SD for continuous variables and as *n* (%) for categorical variables.

**Table 4:** Data from question 18 of the CIS barometer from 1995 to 2012.

Items	1995	1998	2000^*∗*^	2002^*∗*^	2004^*∗*^	2006^*∗*^	2008^*∗*^	2010	2012
Time spent by the physician with you	6.51	6.45	6.39^*∗*^	5.69^*∗*^	6.20^*∗*^	6.23^*∗*^	6.18^*∗*^	6.50	6.72
Number of specialists to whom you have access	7.55	7.62	7.56	6.80^*∗*^	7.40^*∗*^	7.31^*∗*^	7.28^*∗*^	7.47	7.64
Waiting time at the center until seeing the doctor	5.79	5.77	5.62^*∗*^	4.99^*∗*^	5.30^*∗*^	5.32^*∗*^	5.40^*∗*^	5.60	5.72
Knowledge of your medical history and follow-up of your health-related problems	6.71	6.69	6.61^*∗*^	5.86^*∗*^	6.40^*∗*^	6.41^*∗*^	6.41^*∗*^	6.64	6.83
Confidence and trust in your doctor	7.17	7.09	7.08^*∗*^	6.35^*∗*^	6.90^*∗*^	6.90^*∗*^	6.97^*∗*^	7.13	7.29
Easiness to get an appointment	5.49	5.39	5.26^*∗*^	4.89^*∗*^	5.20^*∗*^	5.27^*∗*^	5.32^*∗*^	5.60	5.72
Equipment and technological means available at the center	7.69	7.78	7.69	6.88^*∗*^	7.20^*∗*^	7.20^*∗*^	7.24^*∗*^	7.40	7.54
Manners of healthcare personnel	7.56	7.44	7.37^*∗*^	6.63^*∗*^	7.06^*∗*^	7.11^*∗*^	7.09^*∗*^	7.20	7.42
Information received about your health problem	7.14	7.20	7.16	6.38^*∗*^	6.94^*∗*^	6.94^*∗*^	6.94^*∗*^	7.13	7.30
Medical advice on diet, exercise, smoking, alcohol, and so forth	—	—	—	—	6.60	6.78	6.79	6.98	7.13
Time from medical appointment request to appointment date	—	—	—	—	4.70	4.68	4.67	4.89	4.94
Time taken by the diagnostic tests	—	—	—	—	—	4.73	4.65	4.87	5.04

Sanitary barometer 1995–2012. Healthcare Information Institute. Ministry of Health, Social Services and Equality Madrid, 2013.

Only the year 1995 and years ending in even number are shown.

^*∗*^ Scores lower than preceding years.
